# Label-Free Facial Nerve Identification During Parotidectomy Using Nerve Autofluorescence: An Initial Clinical Series

**DOI:** 10.7759/cureus.112732

**Published:** 2026-07-15

**Authors:** Fernando Dip, Rene Aleman, Alberto Rancati, Federico Marinelli, Javier Ghiselli, Agustina Gorreri, Gustavo Eiben, Diego Sinagra

**Affiliations:** 1 Department of General Surgery, University of Buenos Aires, Buenos Aires, ARG; 2 Heart, Vascular, and Thoracic Institute, Cleveland Clinic Florida, Weston, USA

**Keywords:** facial nerve, fluorescence, intraoperative monitoring, near-ultraviolet imaging, parotidectomy

## Abstract

Facial nerve (FN) dysfunction remains a clinically important complication of parotidectomy, even when recovery is ultimately complete. Traditional nerve identification relies on anatomic dissection, selective stimulation, and, in many cases, intraoperative monitoring (IONM). These approaches may be limited when anatomy is distorted. This study evaluated the feasibility, safety, and utility of autofluorescence-guided FN identification during parotidectomy.

A retrospective case series was conducted in 18 patients who underwent superficial or total parotidectomy for parotid gland tumors. All procedures were performed by the same surgical team using standard operative technique and selective electrical nerve stimulation. Near-ultraviolet (NUV) autofluorescence imaging was obtained immediately after FN exposure and before further dissection. Raw grayscale and real-time colored overlay images were acquired. Quantitative image analysis was performed using manually selected regions of interest to measure nerve and background intensities, nerve-to-background ratio, longitudinal signal homogeneity, and apparent branch width. Given the case series design, findings were summarized using descriptive statistics.

The cohort included 18 patients, 15 women and 3 men, who underwent superficial or total parotidectomy for parotid neoplasms. Final pathology showed pleomorphic adenoma in 15 cases and carcinoma in 3 cases, with deep lobe involvement in 1 case. Autofluorescence imaging generated clear contrast between the FN and surrounding tissue on grayscale and colored overlay views. Mean native grayscale nerve intensities ranged from 244.3 to 247.4 px, compared with background intensities of 41.7 to 45.8 px, yielding nerve-to-background ratios of 5.40 to 5.86. Colored overlay rendering increased the maximum nerve-to-background ratio to 10.16. Longitudinal signal remained uniform, with coefficients of variation (CVs) ranging from 0.028 to 0.043. No postoperative FN paralysis occurred, and all patients had House-Brackmann grade I function at six months.

In this retrospective case series, autofluorescence-guided FN imaging during parotidectomy was feasible and provided consistent visual contrast between nerve and surrounding tissue without identified imaging-related safety concerns. These findings support further study of this approach as a useful adjunct to standard FN identification strategies.

## Introduction

Parotidectomy is a fundamental procedure in head and neck surgery and is most commonly performed for parotid neoplasms, with pleomorphic adenoma representing one of the leading indications for resection in patients with enlarging or symptomatic benign disease [1]. Despite technical refinements, preservation of facial nerve (FN) function remains a central determinant of parotidectomy safety and quality, as postoperative facial weakness continues to be among its most consequential complications [2,3]. In primary parotidectomy, transient FN paresis or paralysis has been reported in 10% to 68% of cases, whereas permanent dysfunction is less common but remains clinically significant, generally occurring in 0.6% to 5%, with higher risk in malignant disease, revision surgery, and more extensive resection [2-4]. Even when temporary, FN dysfunction may produce substantial functional and psychosocial burden through facial asymmetry, impaired expression, ocular protection difficulty, speech and eating limitations, and deterioration in self-image and social confidence [2,3].

Reliable intraoperative identification of the FN is therefore central to parotidectomy. This remains technically demanding because the FN demonstrates meaningful anatomic variability, and tumor size, depth, inflammation, or mass effect may distort its expected relationship to conventional landmarks [5,6]. Anatomical landmarks remain useful, but their reliability may diminish when the gland is expanded or anatomy is displaced, particularly in larger or deep-lobe lesions [5]. Thus, intraoperative monitoring (IONM) has been widely adopted as an adjunct to dissection and nerve confirmation [6,7]. While monitoring provides real-time functional feedback and may assist with localization, aggregate evidence has not shown a consistent or substantial reduction in permanent postoperative FN dysfunction, and any benefit for transient weakness appears variable across studies and clinical settings [6-8].

These limitations support continued interest in technologies that may improve direct intraoperative nerve recognition. Nerve autofluorescence has emerged as a label-free optical approach that permits real-time visualization of neural structures without exogenous contrast administration [4,9]. Early clinical and translational reports suggest that this approach may enhance nerve identification in the operative field, although evidence in parotid surgery remains preliminary [4,9]. Accordingly, this study evaluated the feasibility, safety, and image characteristics of near-ultraviolet (NUV) autofluorescence-guided FN identification during parotidectomy in a clinical case series. The objective was to assess its potential role as an adjunct to standard anatomic dissection and selective nerve stimulation.

## Materials and methods

The study was conducted upon Institutional Review Board approval for retrospective analysis of all patients meeting inclusion criteria. The requirement for informed consent was waived because the study used previously collected, de-identified clinical data, involved no direct patient contact, and did not include any patient intervention. The study was conducted in accordance with the ethical principles of the Declaration of Helsinki. This retrospective single-center case series included consecutive patients who underwent parotidectomy for parotid gland tumors between June 2024 and June 2025. Inclusion criteria were superficial or total parotidectomy for a parotid gland tumor with intraoperative NUV autofluorescence imaging of the FN performed during surgery. Eighteen patients met these criteria and were included for analysis.

**Figure 1:**
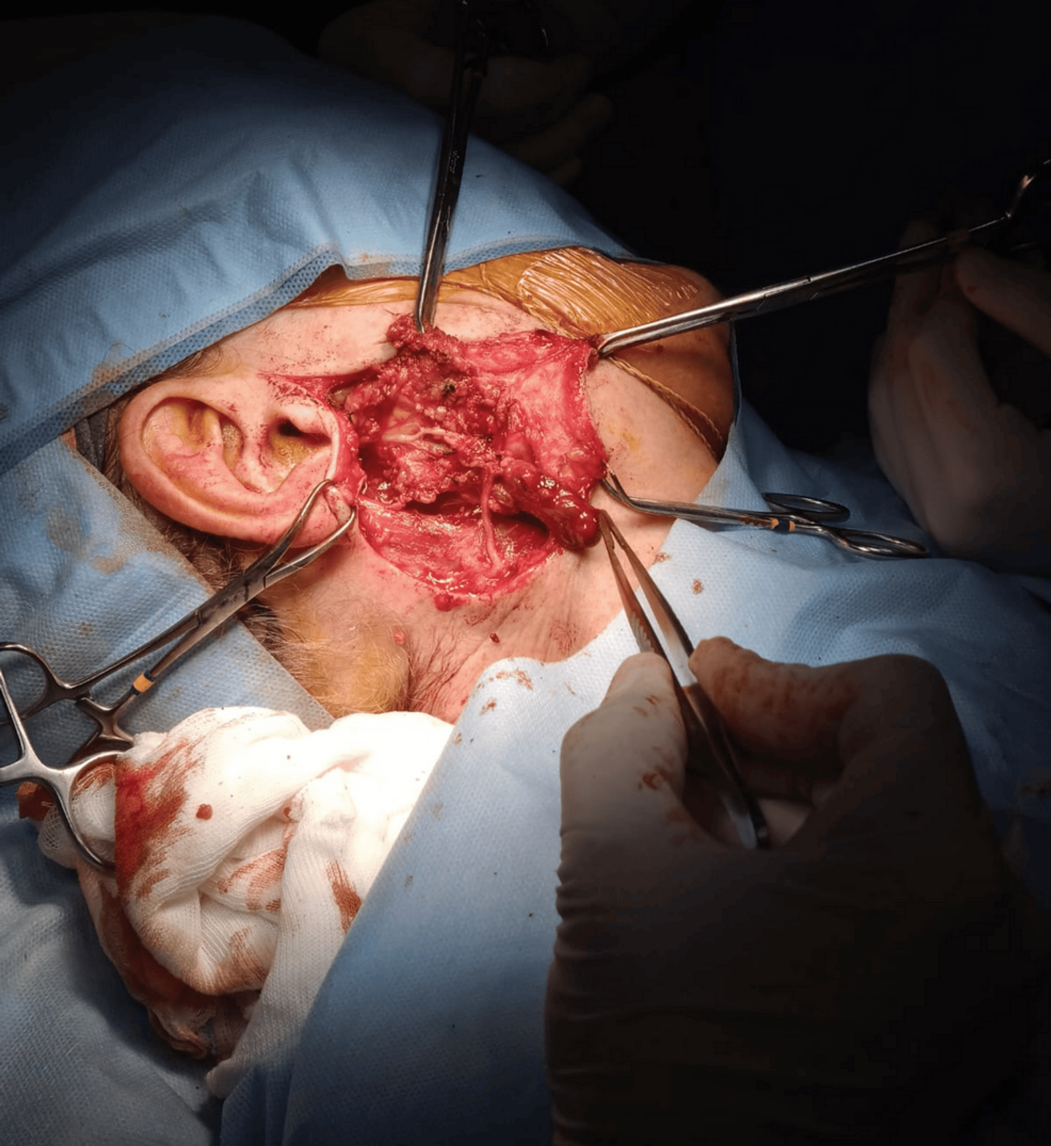
Operative Field After Modified Blair Incision Intraoperative view after a modified Blair incision and elevation of the parotid flap, showing the parotid bed and exposed facial nerve trunk with proximal branching in the auriculotemporal region. This view provides the anatomic context for subsequent NUV autofluorescence imaging. NUV: near-ultraviolet

**Figure 2:**
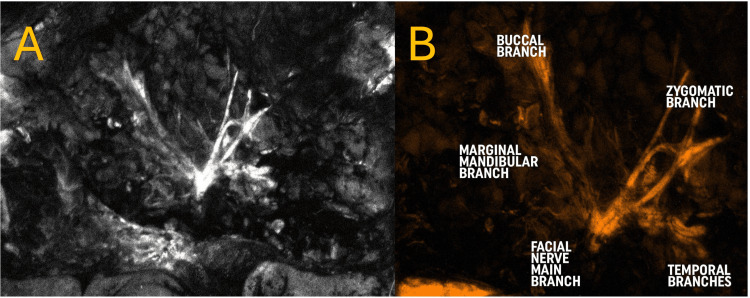
Grayscale and Pseudo-Color Facial Nerve Autofluorescence A) Native grayscale NUV autofluorescence image demonstrating facial nerve arborization within the parotid bed. B) Pseudo-color modality showing enhanced visual contrast of the FN main branch and the distal buccal, zygomatic, marginal mandibular, and temporal branches. NUV: near-ultraviolet; FN: facial nerve

All procedures were performed under general anesthesia by the same surgical team. Standard surgical techniques for superficial or total parotidectomy were used with loupe magnification and selective electrical nerve stimulation (Figure 1). After the FN was initially exposed and identified by the primary surgeon and before further dissection, intraoperative autofluorescence imaging was obtained using the Dendrite® Imaging System, Berkeley, CA, USA (Figure 2). Still images were captured simultaneously in raw NUV grayscale and real-time pseudo-color formats to standardize subsequent image processing. All captured still images were suitable for analysis and were included in the image processing. 

**Figure 3:**
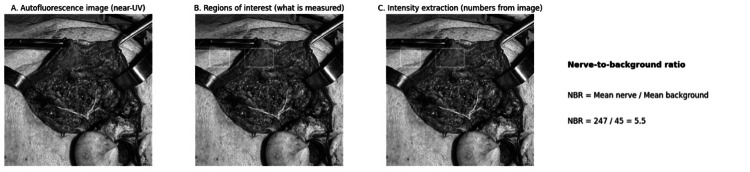
Autofluorescence Signal Analysis Workflow A) Native NUV autofluorescence image of the exposed facial nerve. B) Selected regions of interest used for nerve and background signal extraction. C) Intensity extraction used to calculate the nerve-to-background ratio, defined as the mean nerve intensity divided by the mean background intensity. FN: facial nerve; NBR: nerve-to-background ratio; NUV: near-ultraviolet; ROI: region of interest

Image processing was performed using ImageJ software, version 1.51 (National Institutes of Health (NIH), Bethesda, MD, USA). Processing was performed by three junior surgeons and subsequently reviewed by three senior authors. The FN was isolated as the volume of interest (VOI) relative to adjacent soft tissue and surrounding anatomic structures. Morphometric assessment included nerve length, measured from the most medial captured aspect of the identified neural structure to its most lateral captured aspect, and branching of the main trunk (Figure 3). NUV light intensity distribution histograms and contrast brightness histograms were generated for neural structures in both imaging modalities (e.g., grayscale and pseudo-color formats). Parallel histogram analysis was also performed for adjacent non-neural structures after target isolation to permit descriptive comparison of signal characteristics between the FN and neighboring anatomy. Longitudinal coefficients of variation (CVs) were also calculated along the imaged nerve segment as an exploratory measure of signal homogeneity. These analyses were performed for descriptive and hypothesis-generating purposes only and were not intended to support formal statistical inference. Given the exploratory, noncomparative design and limited sample size, no formal statistical analysis was performed. 

The study objective was to assess the feasibility, safety, and utility of autofluorescence-guided FN identification during parotidectomy. Feasibility was defined as successful intraoperative acquisition of analyzable autofluorescence still images. Safety was assessed by routine postoperative clinical examination of FN function through six-month follow-up. FN function was recorded using the House-Brackmann scale when documented in routine clinical follow-up. Utility was assessed descriptively by intraoperative visualization of the FN in native grayscale and pseudo-color formats and by retrospective image-based morphometric and signal characterization. Given the exploratory, noncomparative design and limited sample size, no formal statistical hypothesis testing was performed. 

Statistical analysis

Given the retrospective, exploratory, and noncomparative case series design, no formal statistical hypothesis testing was performed. Categorical variables were summarized as numbers and percentages. Quantitative autofluorescence parameters were summarized descriptively using mean pixel intensity, nerve-to-background ratio, longitudinal coefficient of variation, 10th percentile, and median branch width, as applicable. All image-based measurements were used for descriptive characterization of autofluorescence signal behavior and were not intended to establish diagnostic thresholds or comparative effectiveness.

## Results

Eighteen patients underwent parotidectomy with intraoperative near-ultraviolet autofluorescence imaging of the FN. Patient and operative characteristics are summarized in Table 1. Final histopathology demonstrated pleomorphic adenoma in 15 patients (83.3%) and carcinoma in three patients (16.7%). One patient (5.6%) displayed a deep-lobe tumor. FN grafting with the sural nerve was required in one case (5.6%). At six-month follow-up, all patients had normal postoperative FN function, with House-Brackmann grade I in 18 of 18 patients (100%). 

**Table 1:** Patient and Operative Characteristics Categorical variables are presented as numbers and percentages, n (%). FN: facial nerve

Characteristic	N = 18, n (%)
Gender	
Male	3 (16.7)
Female	15 (83.3)
Final histopathology	
Pleomorphic adenoma	15 (83.3)
Carcinoma	3 (16.7)
Procedure performed	
Superficial parotidectomy	15 (83.3)
Total parotidectomy	3 (16.7)
Facial nerve function at 6 months - House-Brackmann grade I	18 (100)
Postoperative facial paralysis at 6 months	0 (0%)

Quantitative autofluorescence findings are summarized in Table 2. On native NUV grayscale imaging, mean FN signal intensity ranged from 244.3 to 247.4 px, whereas mean background intensity ranged from 41.7 to 45.8 px. Corresponding nerve-to-background ratios ranged from 5.40 to 5.86. In pseudo-color mode, the maximum nerve-to-background ratio was 10.16, although screen-captured pseudo-color images demonstrated signal saturation. Longitudinal CVs in native images ranged from 0.028 to 0.043. Across the overall imaging analysis, signal variability remained low, with CVs ranging from 0.001 to 0.061. The smallest detectable nerve branches measured 2.8 to 4.4 px in width, and median branch widths ranged from 4.00 to 9.99 px. 

**Table 2:** Quantitative Autofluorescence Metrics From Representative Intraoperative Images Quantitative autofluorescence parameters are presented descriptively as mean pixel intensity, nerve-to-background ratio, longitudinal coefficient of variation, 10th percentile, and median branch width, as applicable. CV: coefficient of variation; FN: facial nerve; NBR: nerve-to-background ratio; NUV: near-ultraviolet; P10: 10th percentile; px: pixels

Image type	Mean nerve intensity (px)	Mean background intensity (px)	NBR	Longitudinal CV	Branch width (px)
NUV grayscale, branch labeled	244.3	41.7	5.86	0.028	p10 4.39, Median: 9.99
NUV grayscale, arborization	247.4	45.8	5.40	0.043	p10: 2.80, Median: 7.19
Pseudo-color visualization	255.0	25.1	10.16	~0.00*	p10: 2.80, Median: 4.00

At six-month follow-up, all patients had normal postoperative FN function, with House-Brackmann grade I in 18 of 18 patients (100%). 

## Discussion

The present study supports the technical feasibility of label-free autofluorescence-guided identification of the FN and its branches during parotidectomy and suggests acceptable short-term safety within the limits of a small, non-comparative experience. The most important finding is not proof of comparative benefit but the demonstration that real-time nerve visualization was achievable across a range of parotid tumors without exogenous contrast administration. In that sense, this study appears to extend the emerging literature from prior mechanistic and early clinical reports [4]. The absence of postoperative facial paralysis at six months in this cohort is reassuring, but it should be interpreted cautiously given the sample size, the low event rate, and the absence of a control group.

Preservation of FN function continues to define the quality and safety of parotid surgery. FN branching is anatomically variable, and contemporary classification work has reinforced that the branching pattern encountered during parotidectomy is often more complex than simplified descriptions suggest, with type I, II, and III patterns reported in 78.26%, 15.22%, and 6.52% of cases, respectively [10]. In parallel, recent outcome data from a tertiary referral cohort of 314 parotidectomies showed that postoperative facial paralysis remains clinically meaningful at 4.4% and was associated with malignant pathology and more extensive surgery, with an association with extensive lymph node dissection (p < 0.001) [11]. Within that operative context, an adjunct that improves visual confidence in trunk and branch recognition is conceptually appealing, particularly when tumor size, depth, or displacement distorts expected anatomy. 

The present findings should also be interpreted in relation to intraoperative FN monitoring rather than against it. Recent evidence suggests that FN monitoring in primary parotidectomy may reduce immediate and permanent postoperative dysfunction across 10 studies including 1,047 patients, of whom 568 underwent monitoring and 479 served as controls [12]. However, monitoring and autofluorescence do not serve the same intraoperative function. Monitoring provides electrophysiologic confirmation, whereas autofluorescence is intended to assist with direct spatial recognition of the nerve within the wound. The quantitative imaging findings in this study, including high nerve-to-background contrast and low longitudinal signal variation, support that optical discriminability was present and remained sufficiently stable to permit branch tracking. Although these image-based measures are exploratory. They do not define a diagnostic threshold, and they do not establish that improved visualization translates into lower rates of traction, thermal, or transection injury.

The biological and translational basis for this approach is supported by earlier autofluorescence work. Peripheral nerve autofluorescence has been characterized ex vivo, then translated to intraoperative human use in an initial clinical series [2,7]. More recently, fluorescence-based recurrent laryngeal nerve visualization during thyroidectomy was reported in 65 cases involving 81 nerves, with all 81 nerves visualized more clearly under NUV light [13]. Taken together with earlier case reporting, these studies suggest that neural autofluorescence is a reproducible optical phenomenon across anatomic sites and operative settings [4]. The present series builds on that foundation by applying the technique to the FN during parotidectomy, where arborization, tissue depth, and tumor-related distortion make continuous visual orientation particularly valuable. 

The pathology distribution in this cohort also matters when interpreting utility. Most cases involved pleomorphic adenoma, a setting in which the operative objective is not only nerve preservation but complete and oncologically sound resection with avoidance of capsular violation. A recent systematic review on pleomorphic adenoma management emphasized recurrence-related lessons tied to surgical strategy and technical error prevention across 15 studies comprising 2,095 patients, with recurrence rates of 3.27% after extracapsular dissection, 0.73% after partial superficial parotidectomy, and 2.41% after superficial parotidectomy [9]. Accordingly, autofluorescence should be viewed as an adjunct to meticulous dissection rather than as a reason to modify established oncologic judgment. Better nerve visualization potentiates ease of dissection, but it does not substitute for the appropriate extent of resection or oncologically oriented tumor handling principles.

Several limitations restrict the strength of inference. This was a retrospective, single-center study with a small sample, no comparator arm, and selection bias because analysis was limited to procedures in which NUV imaging was performed. The cohort was small for meaningful subgroup analysis by histology, extent of parotidectomy, deep lobe involvement, or reconstructive needs. In addition, the study was designed to evaluate feasibility, safety, and image behavior, not comparative effectiveness. Thus, it cannot determine whether autofluorescence is superior to standard dissection, complementary to monitoring in a measurable way, or associated with better patient-centered recovery. Future studies should address said questions prospectively and incorporate standardized functional assessment together with validated patient-reported outcome measures, such as the recently developed Parotidectomy Quality of Life Index (PQLI) [3].

Overall, the present study supports label-free autofluorescence as a practical and compelling intraoperative adjunct for FN identification during parotidectomy. The observed optical performance and favorable short-term clinical experience highlight its potential to enhance real-time nerve visualization without exogenous contrast, offering surgeons an intuitive and field-compatible aid during dissection. These findings provide a strong foundation for continued clinical evaluation and suggest that NUV nerve autofluorescence may become a valuable addition to contemporary nerve preservation strategies.

## Conclusions

In this study, label-free NUV autofluorescence enabled real-time intraoperative visualization of the FN and its branches during parotidectomy without exogenous contrast administration and was associated with favorable short-term FN outcomes. These findings support the feasibility and clinical practicality of this approach as an adjunct for FN identification. Given the small, single-center, non-comparative design, the results should be interpreted as preliminary evidence of feasibility and short-term safety rather than comparative benefit. Prospective comparative studies with standardized FN and patient-reported outcome assessment are needed to define its incremental value in parotid surgery. 
